# A butyrate‐producing synbiotic mitigates intestinal inflammation in a murine colitis model

**DOI:** 10.1002/mlf2.70027

**Published:** 2025-07-30

**Authors:** Hyuna Sung, Soo Yoon Cho, Seong Hyeok Ma, Jin Sun You, Mi Young Yoon, Sang Sun Yoon

**Affiliations:** ^1^ Department of Microbiology and Immunology Yonsei University College of Medicine Seoul Republic of Korea; ^2^ Institute for Immunology and Immunological Diseases Yonsei University College of Medicine Seoul Republic of Korea; ^3^ BioMe Inc. Seoul Republic of Korea

**Keywords:** *Bacillus subtilis*, butyrate, esterase, IBD, tributyrin

## Abstract

Inflammatory bowel disease (IBD) is a chronic condition characterized by intestinal inflammation and gut dysbiosis, with limited treatment options primarily focused on immune‐modulating therapies. Among potential therapeutic agents, butyrate has emerged as a promising candidate due to its anti‐inflammatory and gut‐restorative properties. However, direct administration of butyrate poses significant challenges, including its rapid absorption, uneven distribution within the intestinal tract, and an unpleasant odor that reduces patient compliance. To address these issues, we evaluated the therapeutic potential of *Bacillus subtilis* BM107, a strain selected for its superior butyrate‐producing capabilities and established bacterial safety. BM107 efficiently hydrolyzed tributyrin (TB), a butyrate prodrug, producing substantial butyrate levels in TB‐supplemented media. In a dextran sodium sulfate‐induced colitis mouse model, co‐administration of BM107 and the TB diet significantly improved inflammatory indices, such as reduced disease activity index scores, increased colon length, and restored body weight. Additionally, this combination treatment markedly improved gut microbiome composition, restoring microbial diversity and balance. Furthermore, butyrate levels in the cecum contents of the TB + BM107 group were restored to levels comparable to those in healthy controls, demonstrating the ability of this approach to promote gut homeostasis and intestinal recovery. These findings highlight the therapeutic potential of BM107 combined with a TB diet as a safe, effective, and innovative strategy for addressing gut dysbiosis and inflammation in IBD, paving the way for the development of microbiome‐based bacterial therapeutics to improve patient outcomes.

## Impact statement

This study introduces an innovative synbiotic approach utilizing *Bacillus subtilis* BM107 and tributyrin (TB) to address unmet needs in inflammatory bowel disease (IBD) management. Unlike existing therapies, this strategy leverages BM107's enzymatic properties to enable targeted butyrate production in the gut, bypassing the challenges of direct supplementation. Beyond demonstrating efficacy in reducing inflammation and restoring gut microbiome balance, the study provides a scalable model for integrating microbial therapeutics with metabolic substrates. This research highlights the potential of microbiome‐driven solutions to redefine therapeutic paradigms in IBD, offering new avenues for precision medicine and improved patient outcomes.

## INTRODUCTION

Inflammatory bowel disease (IBD), which includes ulcerative colitis (UC) and Crohn's disease (CD), is a chronic condition characterized by inflammation throughout the gastrointestinal (GI) tract^1^. In recent years, the prevalence of IBD has increased globally, affecting not only the elderly but also younger populations^2^. Common symptoms of IBD include persistent diarrhea, abdominal pain, rectal bleeding, bloody stools, weight loss, and fatigue^3^. This disease profoundly impacts patients' quality of life^4^, and its multifactorial etiology, involving genetic predisposition^5^, environmental triggers^6^, and immune system dysregulation^7^, poses significant challenges for effective treatment.

Short‐chain fatty acids (SCFAs) are carboxylic acids with aliphatic tails containing fewer than six carbons. They are primarily produced through the fermentation of indigestible polysaccharides by gut microbiota^8^. SCFAs play a crucial role in maintaining gut homeostasis by supporting a balanced microbial community, including bacteria, archaea, fungi, and bacteriophages^9^. In the intestinal mucosa, SCFAs exert various beneficial effects on intestinal epithelial cells^10^, including promoting cell proliferation^11^, adenosine triphosphate synthesis in colonocytes, maintaining barrier integrity, and enhancing immunity^12^. Butyrate, one of the most extensively studied SCFAs, is essential for colonic health^13^. It exerts therapeutic effects, particularly through interactions with G protein‐coupled receptors such as GPR43 and GPR109A, which are expressed on colonic epithelial cells. GPR109A, encoded by the *Niacr1* gene, specifically responds to butyrate and niacin in colonic epithelial and immune cells^14^. Activation of GPR109A enhances anti‐inflammatory responses by promoting the activity of colonic macrophages and dendritic cells, further supporting intestinal health^14^. Butyrate also helps differentiate regulatory T cells (Tregs) and interleukin (IL)‐10‐producing T cells, which are essential for maintaining gut homeostasis^15^. Furthermore, butyrate inhibits histone deacetylase, regulating colonocyte proliferation and reducing the risk of colon carcinogenesis^16^, ^17^.

Despite its well‐established benefits, direct supplementation with butyrate presents challenges, such as rapid metabolism, an unpleasant odor, and potential GI side effects. As a result, we believe that a new approach is needed to ensure stable butyrate production in the gut. Tributyrin (TB), a triglyceride prodrug composed of three butyrate molecules esterified to a glycerol backbone, presents a promising alternative^18^. Upon enzymatic cleavage of its ester bonds by host or microbial esterases and lipases, TB releases free butyrate, a SCFA known for its anti‐inflammatory and gut barrier‐protective functions. Through this mechanism, TB alleviates gut dysbiosis and restores intestinal damage in antibiotic‐treated mice^19^. Additionally, TB is a common component of foods, including dairy products, and its slow‐release properties make it a promising candidate for therapeutic applications in IBD and other intestinal disorders^20^. For these reasons, leveraging gut microbes capable of converting TB into butyrate may present a highly effective therapeutic strategy.

*Bacillus subtilis* has a long track record as a probiotic, promoting microbiome restoration and relieving GI symptoms^21^. Because it is a common commensal in the intestinal flora of both humans and animals, the species is regarded as inherently safe^22^. Its value is further enhanced by its robust secretion of hydrolytic enzymes, especially esterases and lipases, that enable the breakdown of a wide array of macromolecules^23^. Given its enzymatic properties, we investigated the potential of *B. subtilis* to increase butyrate levels in the intestinal tract and alleviate inflammatory disease by enhancing the bioavailability of TB through enzymatic response. Here, we propose a novel therapeutic strategy involving a synbiotic agent composed of TB and the probiotic strain *B. subtilis*. This combination could serve as an effective treatment for IBD by enhancing the bioavailability of butyrate, thus supporting gut health and alleviating inflammation.

## RESULTS

### Isolation of TB‐degrading strains

**Figure 1:**
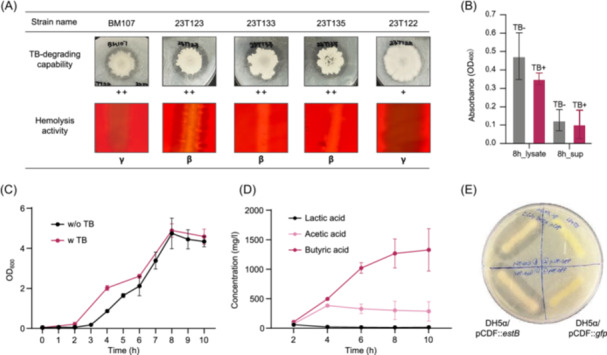
Screening and characterization of tributyrin (TB)‐degrading strains from human feces. (A) *Bacillus subtilis* strains isolated from human fecal samples grown on TB agar plates showing strong TB‐degrading ability. Colonies showing clear zones indicate TB degrading activity (++ for strong activity and + for moderate activity). Hemolysis activity tests were conducted to evaluate bacterial safety, where γ (gamma) indicates nonhemolytic and β (beta) indicates hemolytic activity. Only the nonhemolytic strain BM107 was selected for further analysis. (B) Esterase activity of BM107 measured after 8 h of incubation with and without TB. Cell lysates (8h_lysate) showed higher esterase activity compared to the supernatant (8h_sup), demonstrating intracellular localization of the TB‐degrading enzyme. (C) Growth curve of BM107 cultured in Tryptic Soy Broth with or without TB supplementation. The presence of TB did not significantly affect the growth rate of BM107. (D) Short‐chain fatty acid production during BM107 growth. Butyric acid concentration increased in a time‐dependent manner, indicating efficient TB utilization by BM107. (E) Clear zone formation on TB agar plates by *Escherichia coli* DH5α transformed with the pCDF::*estB* plasmid compared to a negative control with a green fluorescent protein (GFP)‐encoding sequence in place of the *estB* gene (pCDF::*gfp*), confirming the functional role of EstB in TB degradation. The antibiotic resistance tests were conducted three time with indepenent trials.

To isolate strains capable of degrading TB, we developed a selective and sensitive method utilizing TB as a substrate for bacterial butyrate producers. Human fecal microbes were screened on TB agar plates, and strains that formed a clear zone due to TB hydrolysis were selected^24^. As shown in Figure 1A, all the isolated strains showed TB‐degrading activity. Interestingly, upon identification, we found that all selected strains were *B. subtilis*, highlighting this bacterium as a strong candidate for TB degradation. While several other species also formed TB degradation zones, their TB‐degrading ability was much weaker compared to *B. subtilis* (data not shown). As a result, *B. subtilis* strains were chosen for further study.

To assess bacterial safety, we conducted a hemolysis assay with five isolated strains. Among the tested strains, only BM107 and 23T122 showed no signs of β‐ or α‐hemolysis activity on blood agar plates (Figure 1A). Since BM107 showed stronger TB‐degrading activity than 23T122, it was selected as the candidate for the subsequent study. The antibiotic resistance profile of BM107 was evaluated using the E‐test according to Clinical and Laboratory Standards Institute (CLSI) guidelines (Table 1). BM107 showed no resistance to the antibiotics tested, including vancomycin, gentamicin, kanamycin, erythromycin, clindamycin, tetracycline, and chloramphenicol. Although BM107 demonstrated resistance to streptomycin with a minimum inhibitory concentration of 512 µg/ml (Table 1), this resistance is considered an intrinsic property of *B. subtilis* and is not expected to pose any significant safety concerns^25^, ^26^.

**Table 1:** Antibiotic susceptibility test of BM107.

Antibiotic	Cut off (mg/l)	MIC (mg/l)	Result
Vancomycin	4	0.5	S
Gentamicin	4	2	S
Kanamycin	8	6	S
Streptomycin	8	512	R
Erythromycin	4	0.125	S
Clindamycin	4	1.5	S
Tetracycline	8	3	S
Chloramphenicol	8	2	S

MIC, minimum inhibitory concentration; R, resistance; S, sensitive.

### Evaluation of TB utilization and butyrate production by BM107

To further assess the TB‐degrading potential of BM107, we compared its enzymatic activity under culture conditions with or without TB. BM107 was aerobically cultured in Tryptic Soy Broth (TSB) for 8 h, and esterase activity was measured in both the cell lysate and the filtered culture supernatant using 4‐nitrophenyl butyrate (*p*‐NPB) as a substrate. BM107 cell lysate efficiently hydrolyzed *p*‐NPB into 4‐nitrophenolate regardless of the presence of TB, and it showed better hydrolysis in the absence of TB. In contrast, the supernatant of the culture, incubated for the same duration, showed significantly lower hydrolysis compared to the lysate (Figure 1B). These results support the idea that the TB‐degrading enzyme remains in the intracellular space rather than being secreted into the culture medium, and the esterase activity of BM107 is constitutively expressed, whether TB is present or not.

Next, we tested whether BM107 can utilize TB as a carbon source and accumulate its product during growth. Specifically, we aimed to determine if the presence of TB affects BM107 growth, whether butyrate is the final product, or if other metabolites are produced that further influence BM107's growth. To investigate this, we monitored the growth curve and measured changes in SCFAs, including butyric acid and acetic acid, as well as lactic acid, in the culture medium. The growth curve data showed that BM107 grew similarly in the presence of 10 mM TB as it did in the control condition, indicating that TB does not significantly impact its growth (Figure 1C). BM107 began producing butyric acid during the early logarithmic growth phase, reaching its maximum level 8 h post‐inoculation, which was consistent with its growth rate (Figure 1D). The presence of TB and SCFAs did not inhibit BM107's growth in the closed culture system. After 10 h of incubation, BM107 produced 1267.4 ± 246.2 mg/l of butyrate, achieving a conversion rate of approximately 50.2%.

### Genetic characterization and functional validation of BM107's TB catabolic pathway

To gain deeper insights into the genetic traits associated with BM107's TB catabolic ability, we conducted whole‐genome sequencing. The genome analysis revealed that BM107 consists of 4,215,642 bp, with an average G + C content of 43.5%. The genome of BM107 is predicted to encode 4213 protein‐coding genes out of a total of 4331 genes (Table 2). Notably, 24 putative esterases and lipases were identified in its genome, 7 of which are predicted to be involved in TB catabolism based on substrate specificity (Table 3).

**Table 2:** Genome information of BM107.

Feature	Value
Genome size (bp)	4,215,642
G + C content (%)	43.5
Total number of genes	4331
Protein‐coding genes	4213
Average CDS size (bp)	876
rRNA number	30
tRNA number	87
tmRNA number	1

CDS, coding sequence; rRNA, ribosomal RNA; tmRNA, transfer‐messenger RNA; tRNA, transfer RNA.

**Table 3:** Potential enzymes involved in tributyrin catabolism.

No.	Protein name
1	Putative esterase YxiM
2	Carboxylesterase
3	Putative esterase
4	Acylglycerol lipase EstB
5	Putative carboxylesterase nap
6	Carboxylesterase YbfK
7	Lipase EstA

Since TB is an acylglycerol, we postulate that the acylglycerol lipase, EstB, may be responsible for TB breakdown into butyrate. To test this hypothesis, we constructed an *Escherichia coli* strain harboring a plasmid with the cloned *estB* gene. As shown in Figure 1E, the recombinant *E. coli* strain expressing the *estB* gene produced a clear zone on TB‐containing agar plates, demonstrating that the EstB enzyme is primarily responsible for butyrate production. *E. coli* with the control plasmid did not produce a clear zone (Figure 1E). Although *E. coli* has its own native esterases, these were not capable of degrading TB, further supporting the notion that the specific breakdown of TB is due to the enzyme from BM107.

### In vivo therapeutic efficacy of BM107 in a dextran sodium sulfate (DSS)‐induced colitis model

**Figure 2:**
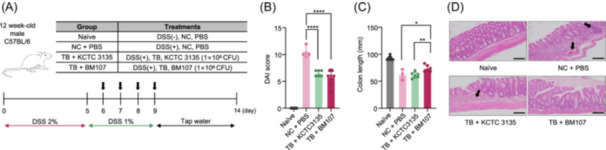
Comparison of therapeutic effects between *Bacillus subtilis* KCTC 3135 and BM107 in a dextran sodium sulfate (DSS)‐induced colitis model. (A) Schematic representation of the experimental design for DSS‐induced colitis in 12‐week‐old male C57BL/6 mice. Mice were divided into four groups: naïve (no DSS, normal chow [NC] diet, phosphate‐buffered saline [PBS] gavage), NC + PBS (DSS, NC diet, PBS gavage), TB + KCTC 3135 (DSS, TB diet, KCTC 3135 gavage), and TB + BM107 (DSS, TB diet, BM107 gavage). Treatments were administered daily from Day 5 to Day 9 as shown. (B) Disease activity index (DAI) scores assessed on the final day (Day 14). Both of TB + BM107 and TB + KCKC 3135 showed significant lower DAI scores compared to the NC + PBS. (C) Colon length measured after mice were killed on Day 14. The TB + BM107 group showed a significant improvement in colon length compared to the NC + PBS and TB + KCTC 3135 groups. (D) Representative hematoxylin and eosin‐stained images of colon tissues from each group. Black arrows indicate regions of tissue damage and inflammatory infiltration. The TB + BM107 group demonstrated greater recovery of colon architecture compared to other groups. Scale bars, 50 μm. The data are presented as means ± standard deviation (SD). All statistical analyses were performed using GraphPad Prism version 9.4.1 (GraphPad Software Inc.). Differences between groups were assessed using a One‐way analysis of variance or Student's *t*‐test. A *p*‐value of <0.05 was considered statistically significant (*p < 0.05; **p < 0.01; ***p < 0.001; ****p < 0.0001).

To investigate the therapeutic potential of BM107 in DSS‐induced colitis, we established a murine model using 12‐week‐old male C57BL/6 mice. As shown in Figure 2A, the mice were treated with 2% DSS for 5 days, followed by 1% DSS for the next 4 days, and then provided with tap water until the end of the experiment (Day 14). The treatment groups included naïve, normal chow (NC) + phosphate‐buffered saline (PBS; DSS‐treated and PBS‐treated), TB + KCTC 3135 (1 × 10^8^ CFU of KCTC 3135), and TB + BM107 (1 × 10^8^ CFU of BM107).

BM107 treatment significantly improved the disease activity index (DAI) score compared to the control groups. As shown in Figure 2B, both the TB + BM107 and TB + KCTC 3135 groups showed a decrease in DAI scores, indicating improvement in colitis symptoms. Colon length is a critical indicator of colitis severity. As shown in Figure 2C, the colon length of the TB + BM107 group was significantly longer compared to the NC + PBS group, further suggesting that BM107 treatment improved intestinal inflammation. The TB + KCTC 3135 group showed a moderate improvement compared to the TB + BM107 group, having a pronounced effect. Representative images of colon tissues stained with hematoxylin and eosin (H&E) are shown in Figure 2D. The colon tissues of the TB + BM107 group showed less severe histopathological changes, such as reduced epithelial damage and inflammation, compared to the NC + PBS group. These findings suggest that the newly isolated BM107 strain demonstrates therapeutic potential comparable to, or potentially superior to, that of previously known *B. subtilis* strains. This highlights its potential for further development and supports the continuation of research to explore its applications.

### Synergistic effects of BM107 and TB co‐treatment in DSS‐induced colitis

**Figure 3:**
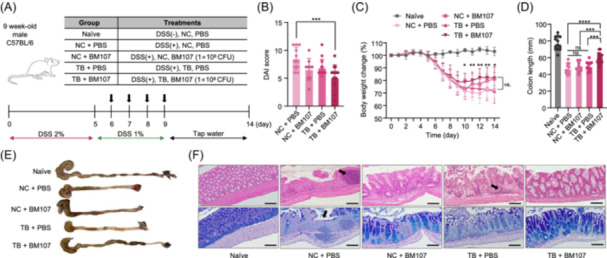
Therapeutic effects of BM107 combined with TB in a DSS‐induced colitis model. (A) Schematic representation of the experimental design for DSS‐induced colitis in 9‐week‐old male C57BL/6 mice. Mice were divided into five groups: naïve (no DSS, NC diet, PBS gavage), NC + PBS (DSS, NC diet, PBS gavage), NC + BM107 (DSS, NC diet, BM107 gavage), TB + PBS (DSS, TB diet, PBS gavage), and TB + BM107 (DSS, TB diet, BM107 gavage). Treatments were administered daily from Day 5 to Day 9. (B) DAI scores lower in the TB + BM107 group compared to the other DSS‐treated groups at Day 14. (C) Changes in body weight during the experimental period presented as percentages of initial body weight. The TB + BM107 group showed less body weight loss compared to the other DSS‐treated groups . (D) Colon length measured after mice were killed, showing significantly longer colons in the TB + BM107 group compared to the NC + PBS and TB + PBS groups. (E) Representative images of excised colons from each group demonstrating improved colon morphology in the TB + BM107 group. (F) Representative histological images of colon tissues stained with hematoxylin and eosin and Alcian Blue‐periodic acid‐Schiff staining (scale bars, 50 μm). Black arrows highlight tissue damage and goblet cell depletion. The TB + BM107 group showed the greatest recovery of tissue structure and goblet cell abundance. Statistical significance was determined using one‐way analysis of variance. *p < 0.05; **p < 0.01; ***p < 0.001; ****p < 0.0001.

BM107 and TB treatment were first assessed for their therapeutic effects in a DSS‐induced colitis model, revealing promising therapeutic potential. However, to obtain more definitive results, we further investigated whether these positive effects were specifically observed only when both the TB diet and the BM107 strain were administered together. To address this, we designed a more refined animal experiment to test the combined effects of TB and BM107 (Figure 3A).

As shown in Figure 3B, treatment with both TB and BM107 together (TB + BM107) resulted in the greatest improvement in DAI scores, demonstrating the most significant reduction in colitis symptoms. While TB alone (TB + PBS) and BM107 alone (NC + BM107) also reduced DAI scores, the combined treatment (TB + BM107) led to a significantly greater improvement compared to either treatment alone. These results highlight that the combination of TB and BM107 provides superior therapeutic efficacy compared to when each is administered separately. The body weight change data shown in Figure 3C further corroborate the findings, with the TB + BM107 group showing the most significant preservation of body weight over the course of the experiment. Interestingly, BM107 alone significantly improved body weight recovery in mice, highlighting its potential as a probiotic even without butyrate supplementation.

The length of the colon was also measured at the end of the experiment. DSS treatment caused a substantial reduction in colon length, the NC + PBS group measuring 32.16% shorter than that of the naïve group (Figure 3D,E). The TB + BM107 group showed significantly longer colon length compared to the NC + PBS, TB + PBS, and NC + BM107 groups (Figure 3D,E). The combination of TB and BM107 treatment was significantly more effective at preserving colon length than either treatment alone, further supporting the superior therapeutic efficacy of the combined approach.

After the mice were killed on Day 14, histological examination of colon tissues revealed significant improvements in the TB + BM107 group. As shown in Figure 3F, the colon morphology of the TB + BM107 group was significantly better than that of the NC + PBS group. The TB + BM107 group showed reduced neutrophil infiltration, as evidenced by arrows in NC + PBS group images (Figure 3F), indicating a reduction in hyper‐inflammatory responses. Moreover, Alcian Blue‐periodic acid‐Schiff (AB‐PAS) staining revealed a substantial recovery of goblet cells in the TB + BM107 group, which are essential for maintaining intestinal barrier function (Figure 3F). The restored mucus production, indicated by the purple and blue areas in the AB‐PAS staining, suggests that TB and BM107 co‐treatment significantly contributes to the recovery of intestinal barrier integrity.

### Restoration of butyrate production and intestinal barrier function by TB and BM107 co‐treatment in DSS‐induced colitis

**Figure 4:**
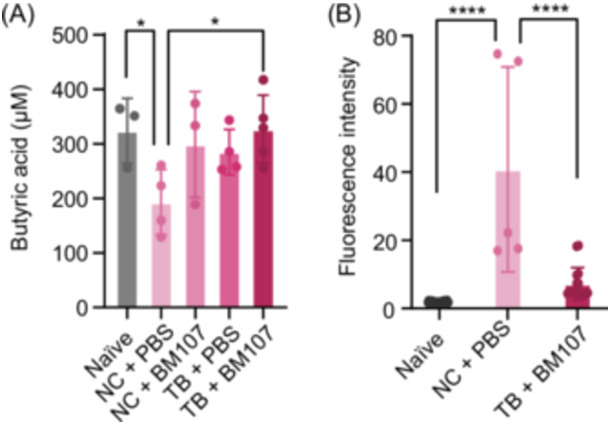
Biochemical assessment of BM107 and TB co‐treatment in a DSS‐induced colitis model. (A) Butyric acid concentrations in the cecum contents of mice. The TB + BM107 group showed significantly restored butyrate levels, comparable to those of the naïve group, highlighting the effectiveness of BM107 and TB co‐treatment (*p < 0.05, *t*‐test). (B) Epithelial barrier integrity assessed using fluorescein isothiocyanate (FITC)‐dextran in serum. The TB + BM107 group demonstrated significantly reduced fluorescence intensity compared to the NC + PBS group, indicating improved epithelial barrier recovery (****p < 0.0001, one‐way analysis of variance).

We hypothesized that co‐administration of TB and the TB‐degrading strain BM107 would generate butyrate and promote intestinal recovery in the DSS‐induced mouse model. To evaluate this, we measured butyrate concentrations using liquid chromatography‐mass spectrometry (LC‐MS) to determine whether they reached levels comparable to those of the naïve group. As shown in Figure 4A, the TB + BM107 group achieved butyrate concentrations similar to those of the naïve group, indicating that the combined treatment was highly effective at restoring butyrate production. In contrast, in the disease condition (NC + PBS group), butyrate synthesis was significantly impaired, demonstrating that colitis induced by DSS treatment results in a severe reduction of butyrate production (Figure 4A). Although the groups treated with BM107 or TB alone had slightly lower butyrate levels than the TB + BM107 group, their recovery was still evident, suggesting that both TB and BM107 could contribute to intestinal healing.

Figure 4B presents the results of the fluorescein isothiocyanate (FITC)‐dextran assay, used to assess intestinal integrity and barrier function. The fluorescence intensity in the TB + BM107 group was significantly lower than that in the NC + PBS group, indicating improved intestinal barrier function in the TB + BM107 group (*p* < 0.0001). Notably, the fluorescence intensity in the TB + BM107 group was comparable to that of the naïve group, suggesting that BM107 treatment restored intestinal barrier function to levels seen in healthy conditions. In contrast, the NC + PBS group showed significantly higher fluorescence intensity compared to other groups, indicating compromised intestinal integrity in the DSS‐induced colitis model. These results underscore the ability of TB + BM107 to restore intestinal barrier function to near‐normal levels, similar to those in the naïve group.

### Impact of TB and BM107 co‐treatment on gut microbiome composition in DSS‐induced colitis

**Figure 5:**
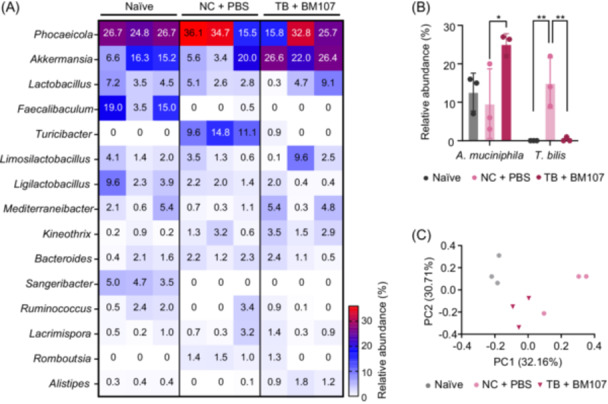
Alterations in gut microbiome composition in a DSS‐induced colitis model. (A) Changes in the microbiome composition of cecum contents analyzed at the genus level across naïve, NC + PBS, and TB + BM107 groups. A heatmap generated using GraphPad Prism illustrates the relative abundance of major genera, with red indicating higher abundance and white indicating lower abundance. (B) Relative abundance of *Akkermansia muciniphila* and *Turicibacter bilis* at the species level. The TB + BM107 group showed significant differences compared to the NC + PBS group (*p < 0.05, **p < 0.01, one‐way analysis of variance). (C) β diversity analysis visualizing gut microbiota compositional differences among naïve, NC + PBS, and TB + BM107 groups. Distinct clustering of microbiota composition was observed between the groups.

DSS‐induced colitis causes alterations in gut microbiome composition, which can serve as indicators of the colitis pathogenesis^27^. Therefore, restoration of the microbiome to its original state may serve as a criterion for assessing disease mitigation^1^, ^28^. To evaluate the effects of the TB diet and BM107 co‐treatment on the gut microbiome under colitis‐induced conditions, we analyzed the cecum microbiome compositions of the NC + PBS group (disease) and the TB + BM107 group, comparing them to the naïve group. The relative abundance of the gut microbiome at the genus level is shown in Figure 5A. The percentages of key genera are as follows: *Bacteroides* (1.4%, 1.9%, and 1.4%), *Phocaeicola* (26.1%, 28.7%, and 24.8%), *Lactobacillus* (5%, 3.5%, and 4.7%), *Limosilactobacillus* (2.5%, 1.8%, and 4.1%), *Turicibacter* (0%, 11.8%, and 0.3%), *Romboutsia* (0%, 1.3%, and 0.4%), *Mediterraneibacter* (2.7%, 0.7%, and 3.5%), *Lacrimispora* (0.6%, 1.4%, and 0.9%), and *Akkermansia* (12.7%, 9.7%, and 25%) across the naïve, NC + PBS (disease), and TB + BM107 groups. The abundance patterns in the TB + BM107 group were similar to those observed in the naïve group, indicating that the microbiome composition in the disease group, which had shifted due to DSS treatment, returned to levels more closely resembling those of the naïve group following TB and BM107 co‐treatment.

Prominent differences at the species level were observed for *Akkermansia muciniphila* and *Turicibacter bilis*. Analysis revealed that *A. muciniphila* and *T. bilis* were the sole species identified within their respective genera (Figure 5A,B). In the TB + BM107 group, the proportion of *A. muciniphila* increased significantly, reflecting recovery similar to that of the naïve group and even exceeding the levels observed in the naïve group. Conversely, *T. bilis* was nearly undetectable in the non inflammatory and recovered groups but constituted a substantial proportion in the NC + PBS group, where inflammation persisted (Figure 5B). Beta diversity, which reflects the differences in microbiome structure between groups, revealed that the microbiome in the TB + BM107 group was much closer to that of the naïve group than to the disease group (Figure 5C). These findings indicate that co‐treatment with TB and BM107 has a significant positive impact on restoring the gut microbiome, highlighting its therapeutic potential in mitigating colitis.

## DISCUSSION

IBD, encompassing conditions such as CD and UC, is a chronic, relapsing inflammatory disorder of the GI tract^1^, ^29^, ^30^, ^31^. The global prevalence of IBD has been steadily increasing, particularly in developed countries, with an estimated 5 million individuals affected worldwide^32^, ^33^. The disease is characterized by periods of active inflammation and remission, with symptoms ranging from abdominal pain, diarrhea, and weight loss to more severe complications like bowel perforation and colon cancer. While the exact etiology of IBD remains unclear, genetic, environmental, and microbial factors are believed to play critical roles in its onset and progression^1^, ^29^.

Current treatment options for IBD primarily focus on managing inflammation and inducing remission through the use of anti‐inflammatory drugs, immunosuppressants, and biologic therapies^1^, ^33^, ^34^. Corticosteroids, aminosalicylates, and immunomodulators are commonly prescribed to control acute flares, while biologics such as tumor necrosis factor (TNF) inhibitors (e.g., infliximab) have revolutionized treatment by targeting specific components of the immune response^31^, ^35^. However, despite these advancements, the management of IBD remains challenging, with many patients either not responding to conventional treatments or experiencing long‐term side effects. Furthermore, current therapies do not address the root causes of the disease, such as the gut microbiome dysbiosis and intestinal barrier dysfunction, and they often come with significant risks, including immunosuppression and increased susceptibility to infections^34^, ^36^.

In light of these limitations, there has been growing interest in microbiome‐based therapies for IBD. The gut microbiome plays a pivotal role in regulating immune responses and maintaining intestinal homeostasis^10^, ^34^, ^37^. Dysbiosis, or an imbalance in the gut microbial community, has been implicated in the pathogenesis of IBD, leading researchers to explore novel therapeutic approaches aimed at restoring a healthy microbiome^38^. Probiotics, prebiotics, fecal microbiota transplantation, and engineered microbiome therapies have emerged as potential strategies to treat IBD by modulating the gut microbiota^37^, ^39^. Among these, microbiome‐based therapeutics, particularly those focusing on restoring microbial balance and enhancing the production of beneficial metabolites like SCFAs, offer promising avenues for treating IBD in a more holistic manner^10^, ^40^. These approaches not only target the symptoms but also aim to restore gut homeostasis, providing a more sustainable and effective solution for managing IBD^28^, ^38^, ^41^.

Butyrate has garnered significant attention as a promising therapeutic agent for various inflammatory diseases, including IBD^13^, ^42^, ^43^. As an SCFA, butyrate plays a crucial role in maintaining gut homeostasis by serving as an energy source for colonocytes, modulating immune responses, and promoting the integrity of the intestinal barrier^13^, ^14^, ^44^. Several studies have demonstrated the beneficial effects of butyrate supplementation in animal models of colitis, where it has been shown to reduce inflammation, enhance mucosal healing, and restore microbial balance^10^, ^43^, ^45^. For instance, butyrate administration has been associated with the inhibition of pro‐inflammatory cytokines, such as TNF‐α and IL‐6, and an increase in anti‐inflammatory markers like IL‐10. These effects are particularly important in IBD, where chronic inflammation disrupts gut function and leads to tissue damage^46^, ^47^.

In clinical settings, various forms of butyrate supplementation, such as butyrate enemas or oral butyrate salts, have been investigated. A study by Steinhart et al. demonstrated that butyrate enemas improved clinical outcomes in patients with UC, including reductions in disease activity and histologic scores^48^. Similarly, De Sabatino et al. reported that oral butyrate supplementation helped alleviate symptoms in patients with CD^42^. Despite these promising results, the direct use of butyrate in clinical practice faces several challenges. One of the major limitations is its rapid absorption in the upper GI tract, which prevents it from reaching the colon in sufficient concentrations to exert therapeutic effects. This challenge is further exacerbated by butyrate's unpleasant odor, which negatively impacts patient compliance.

Moreover, due to its volatile nature, direct butyrate supplementation can lead to unpredictable absorption and bioavailability. These limitations hinder the effective use of butyrate as a treatment for IBD. Therefore, strategies to enhance the localized delivery of butyrate to the colon, such as the use of butyrate‐producing probiotics or butyrate precursors, have become an area of active research. The findings of our study, in which *B. subtilis* BM107, in combination with TB, enhances butyrate production at the site of inflammation, represent a potential solution to the limitations of direct butyrate. By promoting butyrate production locally within the gut, this approach could bypass the need for direct butyrate supplementation, thereby overcoming the issues of rapid absorption and odor while offering sustained therapeutic effects.

Butyrate‐producing bacteria, such as *Faecalibacterium prausnitzii*, *Roseburia* spp., and *Eubacterium rectale*, play a crucial role in gut health by fermenting resistant fibers, such as cellulose, in the diet. However, there are notable challenges in utilizing these naturally occurring butyrate producers to generate the desired quantities of butyrate for therapeutic use^49^, ^50^, ^51^. The most significant hurdle is the need for large amounts of dietary fiber to support sufficient butyrate production. For individuals to generate clinically relevant levels of butyrate, they would need to consume substantial amounts of fiber, which may not be practical or effective for all patients, especially those with dietary restrictions or conditions that impair fiber digestion. Additionally, another major limitation is the variability in the presence of butyrate‐producing bacteria across individuals. The composition of the gut microbiome, including the abundance of butyrate‐producing species, differs significantly from person to person, influenced by factors such as diet, genetics, and environmental exposures. This variability leads to inconsistent butyrate production among different individuals, complicating the standardization of treatments aimed at boosting butyrate levels^52^, ^53^. For example, while some individuals may have a high abundance of *F. prausnitzii*, others may lack key butyrate producers, further complicating the direct use of fiber‐based therapies^54^.

Synbiotics, which combine prebiotics and probiotics, offer synergistic benefits in IBD management^55^. Studies on UC patients utilizing synbiotics, such as *Bifidobacterium* paired with inulin, have demonstrated significant improvements in clinical symptoms, sigmoidoscopy scores, and histological markers, including reductions in TNF‐α and IL‐1α levels^15^, ^38^.

In light of these challenges, a novel synbiotic approach, combining TB as a nutrient substrate with *B. subtilis* BM107, a strain capable of efficiently degrading TB into butyrate, presents an innovative solution. By incorporating both a butyrate precursor and a strain optimized for its production, this approach overcomes the limitations of fiber‐dependent butyrate synthesis. Unlike relying on fiber intake, which requires significant quantities to produce therapeutic amounts of butyrate, the combination of TB and BM107 ensures a more controlled and standardized delivery of butyrate. Furthermore, the ability of BM107 to degrade TB into butyrate in the GI tract offers a reliable method to achieve consistent therapeutic effects, regardless of individual microbiome composition. This synbiotic strategy not only promises to enhance the bioavailability of butyrate but also provides a practical and scalable solution for patients with IBD and other inflammatory diseases, offering a potential breakthrough in microbiome‐based therapies.

In conclusion, our study demonstrates the promising therapeutic potential of a synbiotic approach combining TB and the butyrate‐producing bacterium *B. subtilis* BM107 for the treatment of IBD. The results show that this co‐treatment effectively restores butyrate production in the colon, mitigating inflammation and promoting intestinal recovery in a mouse model of DSS‐induced colitis. By combining TB as a nutrient substrate with BM107, we circumvent the challenges associated with direct butyrate supplementation, such as rapid absorption and unpleasant odor, while ensuring consistent and controlled butyrate production. Furthermore, this approach offers a viable alternative to fiber‐dependent butyrate synthesis, addressing the variability in butyrate‐producing bacteria among individuals. These findings support the potential of TB and BM107 as a novel and scalable synbiotic therapy for IBD, highlighting the therapeutic advantages of microbiome‐based treatments in restoring gut homeostasis and improving patient outcomes.

## MATERIALS AND METHODS

### Isolation and maintenance of BM107

Human feces from a fecal bank were diluted and spread on Tryptic Soy Agar plates supplemented with 2% (v/v) tributyrin (TSB‐TB). After incubating the plates aerobically at 37°C for 24 h, colonies showing a halo around them, indicating TB‐degrading activity, were selected for further study. Detailed procedures have been described elsewhere^56^.

A total of five strains were selected, and all of them were identified as *B. subtilis* through 16S ribosomal RNA sequencing. The pure colonies were cultured in Luria–Bertani (LB) media and preserved in 20% (v/v) glycerol at −80°C. To ensure bacterial safety, hemolysin activity was tested. For administration into the mice, the strains BM107 and *B. subtilis* KCTC 3135 as a counter strain were cultured aerobically in LB broth at 37°C for 20 h with a shaking incubator at 210 rpm and suspended in sterilized 1× PBS to produce 10^8^ CFU/100 μl bacteria. To confirm the bacterial cell concentration, the OD_600_ was measured, and the bacterial suspension was diluted and plated on LB agar for verification.

### Esterase enzyme activity assay

TB esterase activity was assessed using the chromogenic substrate *p*‐nitrophenyl butyrate (*p*‐NPB, Sigma Aldrich), following the methods described previously^57^. The production of *p*‐nitrophenol was measured at 400 nm using a spectrophotometer. For the assay, the isolated bacteria were cultured in LB broth or nonanimal TSB. The cells collected from the cultured broth at various time points were lysed to prepare a lysate. The supernatant obtained by 0.2 µm filtration of the cultured broth was also used as an enzyme source for the assay.

### Whole‐genome sequencing of BM107

HiFi reads generated from the PacBio Sequel IIe system were assembled using the Microbial Assembly Application in SMRTlink 11.1.0.166339, following the Hierarchical Genome Assembly Process 4, with default settings. Illumina raw reads were filtered for quality, retaining those with a Phred score ≥30 for 90% of the bases, and adapters were trimmed using Trimmomatic 0.38. The assembly was then corrected using Pilon v1.21 and high‐quality NovaSeq reads. Assembly quality was assessed with BUSCO. Gene prediction was performed using Prokka v1.14.6, and further annotation was conducted with InterProScan and PSI‐BLAST. Circular contig maps were generated using Circos v0.69.6. The BM107 genome sequence has been deposited to the GenBank (accession code: SAMN46118857).

### Growth curve

All experiments were independently performed in triplicate. BM107 was precultured in 10 ml of LB broth at 37°C for 24 h. The precultured cells were harvested and inoculated into fresh LB broth at an initial OD_600_ of 0.05. At each subsequent cultivation step, the bacterial culture was harvested, adjusted again to an OD_600_ of 0.05, and transferred into fresh 20ml of LB broth. OD_600_ was measured at designated time points during cultivation to determine the growth kinetics.

### Butyrate quantification

The bacterial growth method was the same as described above, with an additional treatment of 10 mM TB in the broth. At each time point, 1 ml of the cultured broth was collected, the cells were pelleted, and the supernatant was filtered using 0.2‐µm pore size syringe filters composed of surfactant‐free cellulose acetate membranes housed in methacrylate butadiene styrene (Minisart® NML, Sartorius). The samples were stored at −20°C until measurement. Butyrate levels were determined using high‐performance liquid chromatography (HPLC) with an Ultimate3000 system (Thermo Dionex), equipped with both a UV detector (210 nm) and a refractive index detector (RefractoMAX520). For analysis, an Aminex 87H column (300 × 10 mm, Bio‐Rad) was used, with 0.01 N H_2_SO_4_ (Fluka) as the eluent. HPLC analysis was performed at the Joint Institute of Agricultural and Life Sciences (NICEM, Seoul National University College of Agricultural and Life Sciences).

### DSS‐induced colitis mouse model

Male C57BL/6 mice, aged 9–12 weeks, were purchased from Orient Bio (Sungnam). The mice were provided with sterile water and food. After a 1‐week adaptation period, the mice were administered 2% DSS (molecular weight, 36–50 kDa; MP Biomedicals) in their drinking water for 5 days, followed by 1% DSS for the next few days, and finally returned to normal drinking water for 5 days. During the study, mice in all groups were fed NC (13.12% energy from fat; PicoLab Rodent Diet 20, LabDiet 5053) for 5 days, after which the diet was switched to a TB diet on the 6th day for the indicated groups, until the day they were killed.

Mice were divided into six experimental groups. The first group (naïve) was fed an NC diet, received no DSS treatment, and was orally gavaged with 100 µl of PBS. The second group (NC + PBS) was fed an NC diet, treated with DSS, and orally gavaged with 100 µl of PBS. The third group (NC + BM107) was fed an NC diet, treated with DSS, and orally gavaged with 100 µl of BM107. The fourth group (TB + PBS) was fed a TB diet, treated with DSS, and orally gavaged with 100 µl of PBS. The fifth group (TB + BM107) was fed a TB diet, treated with DSS, and orally gavaged with 100 µl of BM107. Finally, the sixth group (TB + KCTC 3135) was fed a TB diet, treated with DSS, and orally gavaged with 100 µl of *B. subtilis* KCTC 3135. Following the treatments, including DSS administration and oral gavage with PBS or bacterial suspensions, mice were monitored for body weight and DAI scores. A sample size of at least 10 mice per group was used. Data for body weight and DAI scores included both deceased and surviving mice due to DSS treatment; however, post‐killing experiments were conducted only on surviving mice.

The customized TB diet was prepared by mixing 26.67 ml of glyceryl tributyrate (Sigma‐Aldrich, 91010) into 1 kg of NC diet. DAI scores were recorded based on the modified criteria^58^, as summarized in Table 4. After the mice were killed, the colon was harvested from the cecum to the rectum, measured with a ruler, and divided into thirds. The tissues were opened longitudinally, fixed in 3.7% formaldehyde, paraffin‐embedded, and sectioned at 5 µm. Staining with H&E and AB‐PAS was performed, and the sections were analyzed using an optical microscope (BX53M; Olympus).

**Table 4:** Disease activity index (DAI) scoring for dextran sodium sulfate‐induced colitis.

Score	Decrease in body weight (%)	Stool consistency	Occult/gross rectal bleeding
0	0	Normal	Normal
1	1–6	Lightening or slight looseness	Occult blood +
2	6–11	Lightening and slight looseness	Occult blood ++
3	11–18	Loose stools	Occult blood +++
4	>18	Diarrhea	Gross bleeding

### Intestinal permeability assay using FITC‐dextran

Mice were fasted for 4–6 h before oral administration of FITC‐dextran (4 kDa, Sigma‐Aldrich) to minimize variability in intestinal permeability measurement. During fasting, mice were housed in clean cages without bedding or food, with water provided ad libitum to prevent dehydration. FITC‐dextran was prepared at a concentration of 80 mg/ml in sterile 1× PBS, and each mouse was orally gavaged with 150 µl of the solution. After 4 h, the mice were killed, and whole blood was collected. Serum was separated by centrifugation at 5000 rpm for 10 min at 4°C and transferred to new microfuge tubes. The serum was then loaded into a black opaque‐bottom 96‐well plate, with a PBS blank as a control. Fluorescence intensity was measured using a spectrophotometer, with an excitation wavelength of 485 nm and an emission wavelength of 530 nm.

### LC‐MS analysis of SCFA levels in cecal contents

Butyrate concentrations in cecal contents were quantified using LC‐MS. After mice were killed, cecal samples were collected and immediately processed under 4°C conditions. For each sample, 100 mg of cecal content was homogenized in 1 ml of PBS, followed by centrifugation at 8000 rpm for 15 min. The supernatant was mixed with four volumes of methanol, vortexed, and centrifuged again under the same conditions. The resulting supernatant was collected and stored at −20°C until analysis.

LC‐MS analysis was performed on a Vanquish Flex UHPLC system coupled to a Thermo Scientific instrument using electrospray ionization in negative ion mode. Chromatographic separation was achieved using a Waters Cortecs C8 column (2.1 × 150 mm, 1.6 μm) maintained at 50°C. The mobile phases were 2 mM ammonium acetate in water (A) and methanol (B), with a flow rate of 0.25 ml/min.

Butyric acid was detected with a retention time of 2.05 min and a mass‐to‐charge ratio (*m*/*z*) of 87. A five‐point external calibration curve was constructed using butyric acid standards (20, 50, 100, 200, and 500 μM), demonstrating excellent linearity (*R* ^2^ > 0.999).

### Microbiome composition analysis

DNA was extracted from fecal samples using the DNeasy PowerSoil Kit (Qiagen), following the manufacturer's protocol. The extracted DNA was quantified using the Quant‐IT PicoGreen assay (Invitrogen). Sequencing libraries were prepared according to the Illumina 16S Metagenomic Sequencing Library protocols to target the V3 and V4 regions. For PCR amplification, 2 ng of genomic DNA was used in a reaction mixture containing 5× reaction buffer, 1 mM dNTP mix, 500 nM of each universal forward and reverse primer, and Herculase II fusion DNA polymerase (Agilent Technologies).

The thermal cycling conditions for the first PCR included an initial denaturation at 95°C for 3 min, followed by 25 cycles of 30 s at 95°C, 30 s at 55°C, and 30 s at 72°C, with a final extension at 72°C for 5 min. The universal primers with Illumina adapter overhangs for the first amplification were as follows: V3‐F: 5′‐TCG TCG GCA GCG TCA GAT GTG TAT AAG AGA CAG CCT ACG GGN GGC WGC AG‐3′, V4‐R: 5′‐GTC TCG TGG CTC GGA GAT GTG TAT AAG AGA CAG GAC TAC HVG GGT ATC TAA TCC‐3′. The first PCR product was purified using AMPure beads (Agencourt Biosciences). For final library construction, 2 μl of the purified first PCR product was subjected to a second round of PCR using NexteraXT Indexed Primers under the same conditions as those of the first PCR but with 10 cycles. The final PCR products were purified with AMPure beads.

Quantification of the purified libraries was performed using qPCR with the KAPA Library Quantification kits for Illumina Sequencing platforms, and quality assessment was performed using the TapeStation D1000 ScreenTape (Agilent Technologies). Paired‐end sequencing (2 × 300 bp) was conducted by Macrogen on an Illumina MiSeq platform (Illumina).

### Statistical analysis

The data are presented as means ± standard deviation (SD). All statistical analyses were performed using GraphPad Prism version 9.4.1 (GraphPad Software Inc.). Differences between groups were assessed using a one‐way analysis of variance or Student's *t*‐test. A *p*‐value of <0.05 was considered statistically significant (*p < 0.05, **p < 0.01, ***p < 0.001, and ****p < 0.0001).

## AUTHOR CONTRIBUTIONS

**Hyuna Sung**: Conceptualization; data curation; formal analysis; funding acquisition; investigation; methodology; writing—original draft; writing—review and editing. **Soo Yoon Cho**: Data curation. **Seong Hyeok Ma**: Methodology. **Jin Sun You**: Investigation. **Mi Young Yoon**: Resources; supervision. **Sang Sun Yoon**: Conceptualization; writing—review and editing.

## ETHICS STATEMENT

All animal experiments in this study were performed in compliance with the guidelines established by the Department of Animal Resources at the Yonsei Biomedical Research Institute. This study was approved by the Institutional Animal Care and Use Committee (IACUC) of Yonsei University College of Medicine under permit numbers 2024‐0143 and 2021‐0139.

## CONFLICT OF INTERESTS

The authors declare no conflict of interests.

## Data Availability

All data generated or analyzed during this study are included in this article.
